# The loss of succinate dehydrogenase B expression is frequently identified in hemangioblastoma of the central nervous system

**DOI:** 10.1038/s41598-019-42338-z

**Published:** 2019-04-10

**Authors:** Tae Hoon Roh, Hyunee Yim, Jin Roh, Kyi Beom Lee, So Hyun Park, Seon-Yong Jeong, Se-Hyuk Kim, Jang-Hee Kim

**Affiliations:** 1 Department of Neurosurgery, Ajou University School of Medicine, Suwon, 16499 Korea; 2 Department of Pathology, Ajou University School of Medicine, Suwon, 16499 Korea; 3 Department of Medical Genetics, Ajou University School of Medicine, Suwon, 16499 Korea

## Abstract

Succinate dehydrogenase (SDH) is a mitochondrial enzyme that plays an important role in both the Krebs cycle and the electron transport chain. SDH inactivation is associated with tumorigenesis in certain types of tumor. SDH consists of subunits A, B, C and D (SDHA, SDHB, SDHC, and SDHD, respectively). Immunohistochemistry for SDHB is a reliable method for detecting the inactivation of SDH by mutations in *SDHA*, *SDHB*, *SDHC*, *SDHD* and *SDH complex assembly factor 2* (*SDHAF2*) genes with high sensitivity and specificity. SDHB immunohistochemistry has been used to examine the inactivation of SDH in various types of tumors. However, data on central nervous system (CNS) tumors are very limited. In the present study, we investigated the loss of SDHB immunoexpression in 90 cases of CNS tumors. Among the 90 cases of CNS tumors, only three cases of hemangioblastoma showed loss of SDHB immunoexpression. We further investigated SDHB immunoexpression in 35 cases of hemangioblastoma and found that 28 (80%) showed either negative or weak-diffuse pattern of SDHB immunoexpression, which suggests the inactivation of SDH. Our results suggest that SDH inactivation may represent an alternative pathway in the tumorigenesis of hemangioblastoma.

## Introduction

Succinate dehydrogenase (SDH) is an important mitochondrial enzyme that participates in the Krebs cycle and the electron transport chain^1,2^. It consists of four subunits: SDHA, SDHB, SDHC, and SDHD. Each subunit is encoded by the corresponding *SDHA*, *SDHB*, *SDHC*, and *SDHD* gene in the nucleus and is incorporated at the inner mitochondrial membrane. A functional unit, SDH complex assembly factor 2 (SDHAF2), which is encoded by the *SDHAF2* gene, is also required for its enzymatic activity^2–4^.

Interestingly, in addition to its pivotal role in normal aerobic respiration, SDH has tumor-suppressive effects^5–7^. SDH inactivation results in the accumulation of succinate and induces the stabilization of hypoxia-inducible factor (HIF) via competitive inhibition of HIF prolyl-hydroxylases. Stabilized HIF activates pseudo-hypoxic signaling and leads to angiogenesis, the dysregulation of cellular proliferation, and adhesion^5,8–11^. The accumulation of succinate may also be associated with alteration of epigenomic landscapes favoring oncogenesis through the inhibition of histone demethylation^12^.

The inactivation of SDH can be caused by any mutation of *SDHA*, *SDHB*, *SDHC*, *SDHD*, or *SDHAF2* (*SDHx* genes)^2–4^. Germline mutations in *SDHx* genes were first believed to be limited to familial paraganglioma/pheochromocytoma^7^. However, it has since been reported in other solid tumors, such as gastrointestinal stromal tumors (GISTs)^13,14^, renal cell carcinomas (RCCs)^15–17^, pituitary adenomas (PAs)^18–20^, and pancreatic neuroendocrine tumors (NETs)^19^.

Immunohistochemistry for SDHB is a reliable method for detecting *SDHx* mutations with high sensitivity and specificity^21–24^. Various types of tumors have been evaluated to determine the status of *SDHx* mutations using SDHB immunohistochemistry^15,19,21–27^. However, data on *SDHx* mutations of central nervous system (CNS) tumors are very limited^19,28,29^. Furthermore, to the best of our knowledge, the loss of SDHB immunoexpression has not been explored in various types of CNS tumors.

In the present study, we performed SDHB immunohistochemistry in various types of CNS tumors and found a significant proportion of hemangioblastomas with loss of SDHB immunoexpression.

## Results

### SDHB immunohistochemistry in CNS tumors

**Figure 1:**
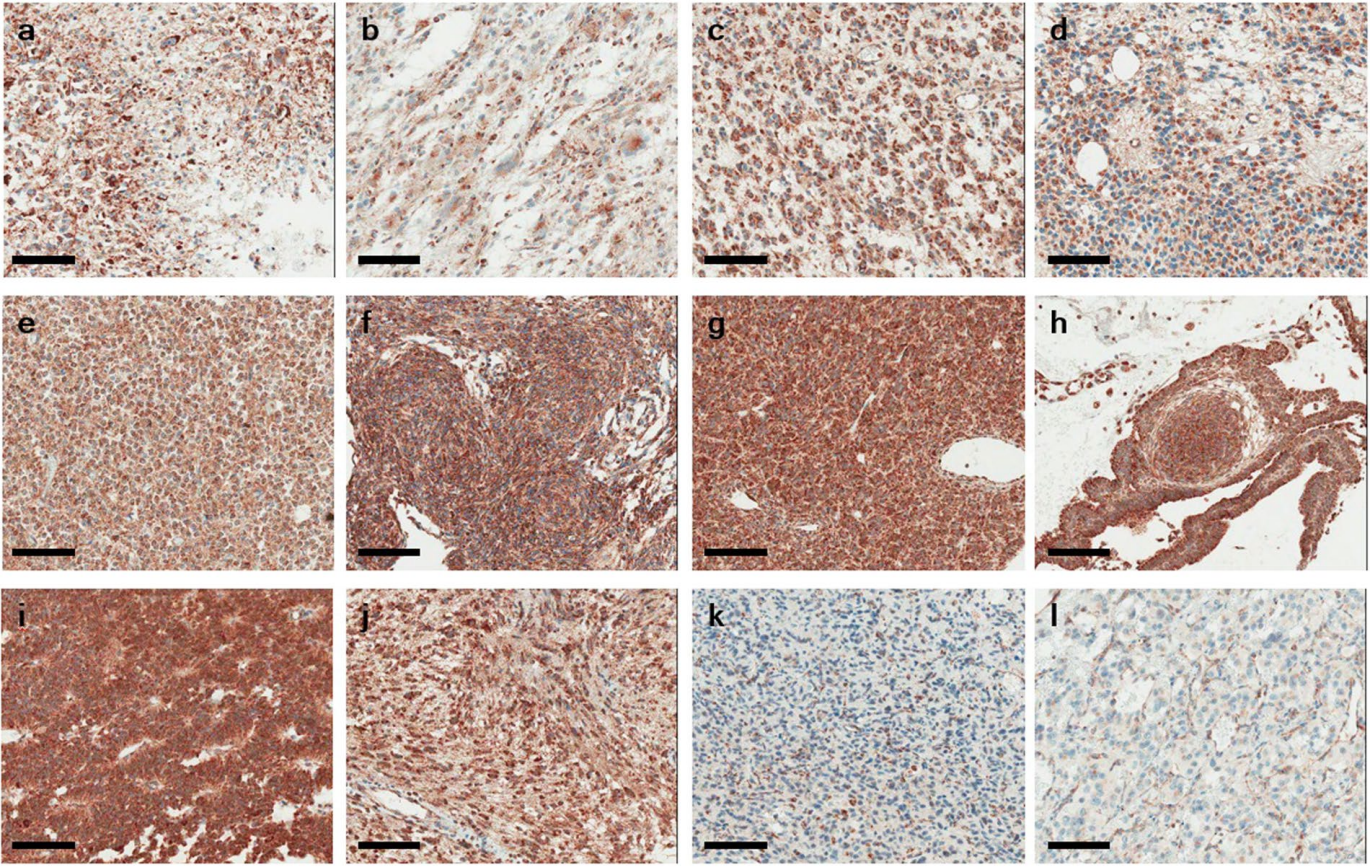
SDHB immunohistochemistry showed strong granular cytoplasmic positivity in central nervous system tumors except for hemangioblastoma and control pheochromocytoma with an SDHB mutation. (**a**) Glioblastoma, (**b**) Astrocytoma, (**c**) Oligodendroglioma, (**d**) Ependymoma, (**e**) Central neurocytoma, (**f**) Meningioma, (**g**) Hemangiopericytoma, (**h**) Craniopharyngioma, (**i**) Pituitary adenoma, (**j**) Schwannoma, (**k**) Hemangioblastoma, (**l**) Control: Pheochromocytoma with an SDHB mutation. Bar indicates 100 µm.

To screen for the inactivation of SDHB across CNS tumors, we performed SDHB immunohistochemistry using TMA blocks including 17 cases of glioblastoma, 7 of astrocytoma, 9 of oligodendroglioma, 9 of ependymoma, 10 of meningioma, 6 of hemangiopericytoma, 7 of central neurocytoma, 12 of PA, 5 of craniopharyngioma, 3 of schwannoma, and 3 of hemangioblastoma. In all, 81 cases (90%) of CNS tumors showed positive staining for SDHB in the whole tumor, and 6 cases (6.7%) revealed strong granular SDHB immunoreactivity in part of the tumor area. Among the 90 cases of CNS tumors, only 3 (3.3%) showed no immunoexpression of SDHB protein (Table 1). Interestingly, all three cases were hemangioblastoma (Fig. 1).

**Table 1:** SDHB immunonegativity in 90 cases of central nervous system tumors.

Tumor type	SDHB immunonegativity
Glioblastoma	0/17
Astrocytoma	0/7
Oligodendroglioma	0/9
Ependymoma	0/9
Central neurocytoma	0/7
Schwannoma	0/3
Meningioma	0/10
Hemangiopericytoma	0/6
Hemangioblastoma	3/3
Craniopharyngioma	0/5
Pituitary adenoma	0/12

### SDHB immunohistochemistry in hemangioblastomas

**Figure 2:**
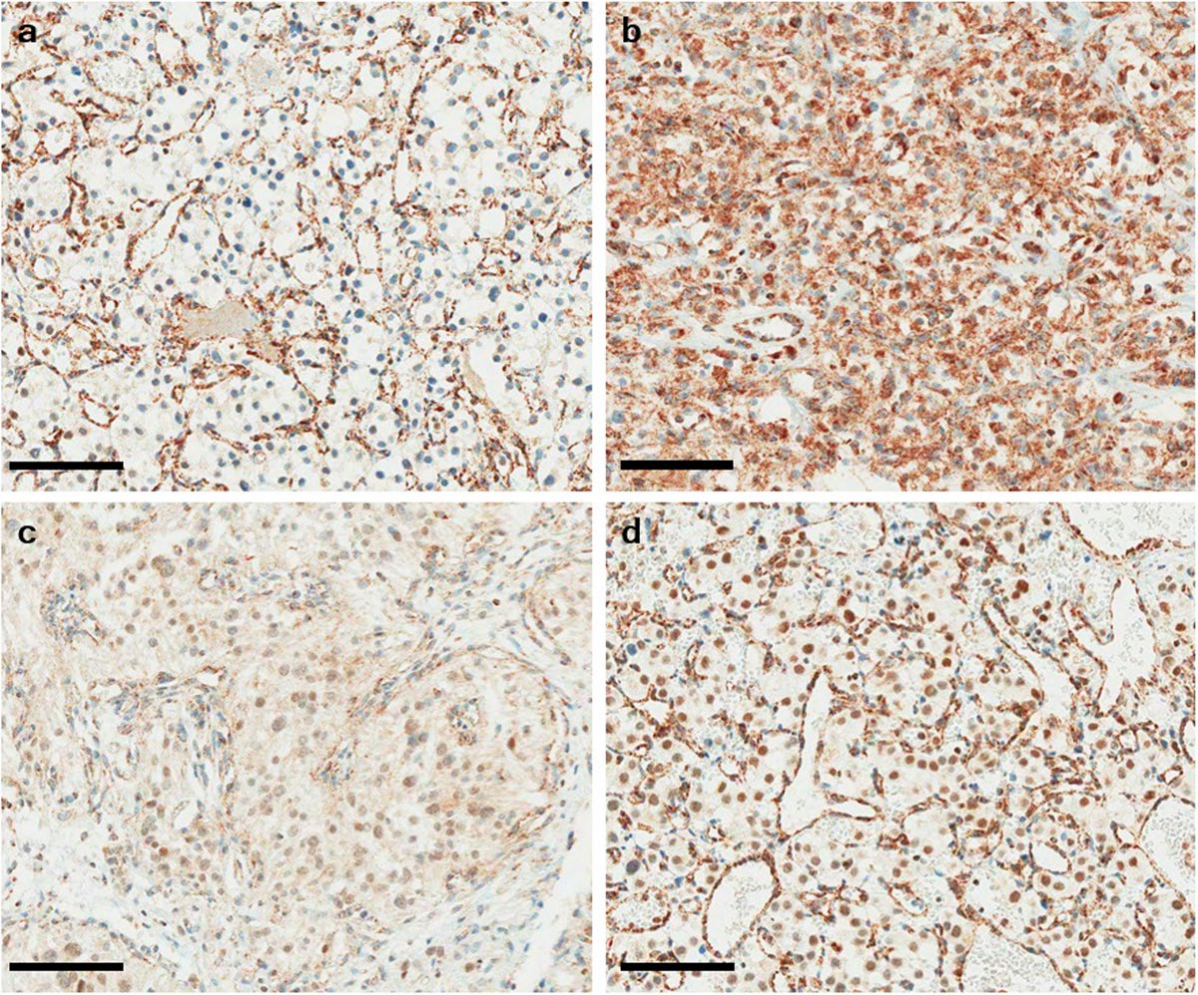
SDHB immunohistochemistry of hemangioblastoma. (**a**) Hemangioblastoma with SDHB immunonegativity shows no immunoexpression in the cytoplasm in the presence of strong granular staining of capillary endothelial cells (internal control). (**b**) Hemangioblastoma with strong granular cytoplasmic positivity. Hemangioblastoma with a weak-diffuse pattern of SDHB immunoexpression showing mild cytoplasmic (**c**) and/or nuclear blush staining (**d**). Bar indicates 100 µm.

**Figure 3:**
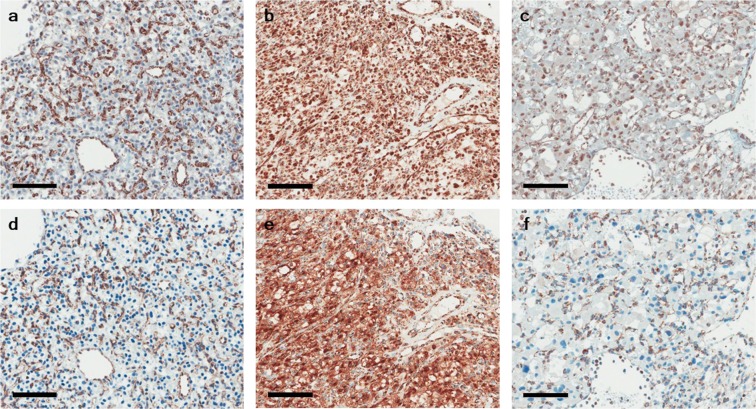
SDHB immunohistochemistry of hemangioblastoma with two different primary antibodies. (**a**) Hemangioblastoma with SDHB immunonegativity, (**b**) strong granular cytoplasmic positivity, and (**c**) a weak-diffuse pattern and/or nuclear blush staining using a primary rabbit polyclonal antibody (HPA002868). (**d**–**f**) reveal results of SDHB immunostaining with a different primary mouse monoclonal antibody (ab14714). SDHB expression patterns of (**d**,**e**) are consistent with those of corresponding (**a**,**b**) areas, respectively. (**f**) show negative SDHB staining. Bar indicates 100 µm.

To examine SDHB immunoexpression in hemangioblastoma, we performed SDHB immunohistochemistry in 35 hemangioblastoma cases with two different primary antibodies against SDHB. The clinical characteristics of the 35 patients with hemangioblastoma are summarized in Table 2. First, we performed SDHB immunohistochemistry with a primary rabbit polyclonal antibody (HPA002868). Among the 35 cases, 9 (25.7%) showed negative staining for SDHB, whereas 7 (20%) showed strong granular staining in the cytoplasm. Among the seven cases of strong granular positivity, two showed partial loss of SDHB. The remaining 19 cases (54.3%) revealed a week-diffuse pattern of SDHB immunostaining (Fig. 2) (Table 3). Next, we performed an additional SDHB immunohistochemistry with the different primary mouse monoclonal antibody (ab14714) and compared the results of both SDHB immunostainings. SDHB expression patterns in 9 of negative and 7 of strong granular staining were almost identical in both SDHB immunostainings. However, among 19 cases of a week-diffuse pattern of SDHB immunostaining with a primary rabbit polyclonal antibody, only 10 (28.6%) revealed a week-diffuse pattern of SDHB immunostaining, whereas remaining 9 (25.7%) showed negative staining (Fig. 3) (Table 3).

**Table 2:** Clinical characteristics of 35 patients with hemangioblastoma.

Male:Female	25:10
Mean age (years ± SD)	41 ± 8.5
**Tumor location**	
Cerebellum (%)	29 (82.9)
Spinal Cord (%)	6 (17.1)
**Association with VHL**	
Sporadic (%)	33 (94.3)
VHL (%)	2 (5.7)

**Table 3:** Results of SDHB immunohistochemical staining in 35 cases of hemangioblastoma.

Case no	Sex	Age	Location	SDHB (HPA002868)	SDHB (ab14714)
1	M	58	Cerebellum	Strong	Strong
2	F	47	Cerebellum	Negative	Negative
3	M	25	Cerebellum	Weak-diffuse	Weak-diffuse
4	M	27	Cerebellum	Weak-diffuse	Weak-diffuse
5	M	42	Cerebellum	Negative	Negative
6	M	41	Cerebellum	Weak-diffuse	Negative
7	M	23	Spinal cord	Weak-diffuse	Negative
8	M	55	Cerebellum	Negative	Negative
9	M	41	Spinal cord	Negative	Negative
10	M^†^	30	Cerebellum	Weak-diffuse	Weak-diffuse
11	F	29	Cerebellum	Strong	Strong
12	M	35	Cerebellum	Strong	Strong
13	M	26	Cerebellum	Weak-diffuse	Negative
14	M	48	Cerebellum	Weak-diffuse	Negative
15	F	36	Spinal cord	Weak-diffuse	Negative
16	M^†^	30	Cerebellum	Weak-diffuse	Weak-diffuse
17	M	46	Spinal cord	Negative	Negative
18	M	15	Spinal cord	Weak-diffuse	Weak-diffuse
19	M	39	Cerebellum	Strong	Strong
20	F	75	Cerebellum	Weak-diffuse	Weak-diffuse
21	F	53	Cerebellum	Weak-diffuse	Weak-diffuse
22	F	15	Cerebellum	Weak-diffuse	Negative
23	M	42	Cerebellum	Negative	Negative
24	F	32	Cerebellum	Negative	Negative
25	M	51	Cerebellum	Weak-diffuse	Weak-diffuse
26	M	54	Cerebellum	Strong^‡^	Strong^‡^
27	F	69	Cerebellum	Weak-diffuse	Negative
28	M	56	Cerebellum	Weak-diffuse	Weak-diffuse
29	M	44	Cerebellum	Negative	Negative
30	M	44	Cerebellum	Negative	Negative
31	M	22	Spinal cord	Strong	Strong
32	F	32	Cerebellum	Weak-diffuse	Weak-diffuse
33	M	37	Cerebellum	Weak-diffuse	Negative
34	F	51	Cerebellum	Weak-diffuse	Negative
35	M	71	Cerebellum	Strong^‡^	Strong^‡^

^†^VHL-associated case. ^‡^Cases showing strong granular SDHB immunostaining with partial negative staining.

### Mutation analyses in hemangioblastomas

We performed mutational analyses by direct sequencing in 10 cases. Among 10 cases, 4 cases were negative for SDHB immunostaining and remaining 6 were cases with a weak-diffuse pattern of SDHB immunostaining. We did not detect any pathogenic *SDHB* mutations except for three cases of a mutated exon 4 and one case of a mutated exon 1, which failed to amplify. Interestingly, we found an SDHB c.18C > A single nucleotide variant in all nine cases of hemangioblastoma, which was present in exon 1. We did not observe any pathogenic mutations in previously reported missense mutation sites of SDHA (Chr5:254599, Chr5:256509, or Chr5:223646 on Assembly GRCh37)^30^.

## Discussion

SDH was the first mitochondrial enzyme identified as a tumor suppressor^5,7^. Among the SDH complex, SDHA and SDHB are hydrophilic catalytic subunits, whereas SDHC and SDHD are hydrophobic and anchor the catalytic subunits to the inner mitochondrial membrane^2–4^. If any component of the SDH complex is lost, SDHB protein is released into the cytoplasm and rapidly degraded^3,4,24^. Remarkably, SDHB immunohistochemistry shows negative immunoexpression in the presence of bi-allelic inactivation of any of *SDHx* mutation and has been suggested to be a surrogate marker for *SDHx* mutation^21–24^. Because of its wide expression and fundamental role in cell biology, its inactivation may be associated with other neoplasms beyond paraganglioma/pheochromocytoma^3,5,8,31^. Various types of tumors were reported in *SDHx* mutation carriers^14,15,17,20,25,31^ or have been identified in a series of tumors that have not been genetically characterized^19,27,31^. SDHB immunonegativity has been reported in pheochromocytoma/paraganglioma, GISTs, RCCs, PAs, pancreatic NETs, prostate cancer, stomach cancer, and testicular seminoma^15,20,22–24,27,31^. However, data on CNS tumors are very limited. A retrospective cohort study on *SDHx* mutation carriers^19^ reported a case of meningioma in a patient with *SDHA* germline mutation and a case of oligodendroglioma in a patient with *SDHD* germline mutation. However, these tumors showed positive SDHB immunoexpression, which suggests the absence of SDH inactivation. A case of atypical meningioma was reported in a patient with a germline mutation in the *SDHB* gene and molecular analyses with tumor tissue confirmed an *SDHB* mutation in the meningioma. However, SDHB immunohistochemistry was not performed^29^. A recent study suggested that oligodendrogliomas with a 1p19q deletion are associated with the downregulation of SDHB expression, but SDHB immunohistochemistry was not performed^28^. In the present study, we performed SDHB immunohistochemistry on various types of CNS tumors and observed that all cases of oligodendroglioma (9 cases) and meningioma (10 cases) showed strong granular immunopositivity. Previous studies have reported that PA may harbor mutations in *SDHx* genes and exhibit SDHB immunonegativity^18,20^. However, SDH inactivation in PA is very rare (only 0.3%)^4,32^. In the present study, we did not detect any loss of SDHB immunoexpression in PAs. Unexpectedly, we found SDHB immunonegativity in hemangioblastoma.

Hemangioblastomas arising in the CNS are benign tumors composed of large and vacuolated stromal cells and numerous thin-walled blood vessels. CNS hemangioblastomas most often occur in the cerebellum, followed by the brainstem and spinal cord^33,34^. Approximately 25% of hemangioblastomas are associated with von Hippel-Lindau (VHL) disease, whereas the remaining 75% of cases are sporadic^33^. In VHL-related hemangioblastomas, bi-allelic inactivation of the *VHL* gene can induce HIF stabilization. As a result, HIF induces the activation of genes related to the tumorigenesis of VHL disease^33,35,36^. Recent studies have suggested that the inactivation of VHL plays a dominant role not only in the pathogenesis of familial hemangioblastomas but also in the sporadic form^30,37^. However, a significant proportion of sporadic hemangioblastomas still exist without VHL inactivation, which suggests that alternative pathways may be involved in the tumorigenesis of sporadic hemangioblastomas^37^. In the present study, 80% of hemangioblastomas showed either negative or weak-diffuse pattern of SDHB immunoexpression, which suggests the inactivation of SDH. Clinical manifestations of the *SDHx* mutation are very similar to those of VHL disease. Paraganglioma/pheochromocytoma, RCC, and pancreatic NET can be caused by both disease entities^3,4,19,31,35,36^. Furthermore, the inactivation of SDH and VHL can share a common pathway via HIF stabilization^5,10,11,38^. Therefore, our results suggest that SDH inactivation may represent one alternative pathway involved in the tumorigenesis of sporadic hemangioblastoma.

SDH inactivation-related tumors can be caused irrespective of the type of *SDHx* mutation. However, there are some correlations between the tumor type and mutation frequency. *SDHB* and *SDHD* mutations are common in pheochromocytoma/paraganglioma^19,24,38^, and *SDHA* mutations and *SDHC* promoter hypermethylation are relatively common in GISTs^4^. In RCC, *SDHB* mutations are more common^3,4,31^. PA is frequently associated with *SDHA* mutations^4^. Although several recent studies have performed comprehensive molecular analyses on a series of hemangioblastomas^30,39,40^, data related to *SDHx* mutations in hemangioblastoma are rare. Shankar *et al*. performed molecular analyses of hemangioblastomas using deep-coverage DNA sequencing. They reported inactivation of the *VHL* gene in 78% of sporadic hemangioblastomas, but no other gene was significantly mutated^30^. In the supplementary data of that study, we found seven cases of hemangioblastoma with missense mutations in *SDHA* and *SDHB* genes. However, missense mutations in the *SDHA* gene were benign or uncertain significance and only one mutation in the *SDHB* gene was associated with conflicting interpretations of pathogenicity. In the present study, we performed direct sequencing on whole exons of the *SDHB* gene and on previously reported missense mutation sites in the *SDHA* gene^30^. However, we did not find any pathogenic mutation. We further performed SDHA immunohistochemistry, but did not observe any loss of SDHA immunoexpression (Supplementary Fig. S1). Only one type of *SDHB* polymorphism was found (c.18C > A single nucleotide). This polymorphism is one of well-known polymorphisms and found with a frequency of 2.7% in Danish patients with neuroendocrine cancer^41^. In the present study, because of our limited analytical methods, we could not demonstrate an association between SDHB immunonegativity and causal mutations in *SDHx* genes. Therefore, to elucidate how the inactivation of SDHB is related to mutations in *SDHx* genes, further comprehensive genomic studies, including epigenomic analyses of *SDHx* genes, is needed.

SDHB immunohistochemical results should be interpreted with caution, because false negative immunostaining may be associated with tissue quality, poor fixation, and/or immunohistochemical technique. Therefore, it is important to use an internal positive control in non-neoplastic cells, such as endothelial, stromal, or inflammatory cells, throughout the tumor before interpreting SDHB immunohistochemistry. If there is no internal positive control, SDHB staining should not be interpreted irrespective of the status of SDHB immunoexpression in the tumor^4,23,24^. In the present study, all 35 cases of hemangioblastoma were compared to the internal positive control. SDHB immunostaining must be considered positive (normal) when strong granular cytoplasmic positivity (mitochondrial pattern) is present. However, the interpretation of a weak-diffuse staining pattern can be challenging^4,22,23^. This pattern has been reported 3.7–11.5% of paragangliomas/pheochromocytomas and was frequently identified in cases with *SDHD* and *SDHB* mutations^4,22–24^. Therefore, a week-diffuse pattern of SDHB immunostaining, particularly when this contrasts markedly with true mitochondrial (strong granular cytoplasmic) staining in internal positive controls, should correctly be considered negative and indicative of SDH deficiency^4,22–24^. In the present study, we initially observed a weak-diffuse pattern of SDHB in 19 cases (54.3%) of hemangioblastoma. However, an additional SDHB immunohistochemistry with a different antibody showed only 10 (28.6%) of them were a weak-diffuse pattern. Our results suggest that an additional SDHB immunohistochemistry with a different antibody could be beneficial when SDHB immunostaining showed a weak-diffuse pattern and support the idea that a week-diffuse pattern of SDHB immunostaining, in internal positive controls, should correctly be considered negative and indicative of SDH deficiency^4,22–24^. Since the inactivation of SDH involves the HIF-1α pathway^5,10,11,38^, we additionally performed HIF-1α immunostaining with 9 cases of a weak-diffuse pattern of SDHB and found that all of the cases showed revealed an increased expression of HIF-1α suggesting the inactivation of SDH (Supplementary Fig. S2).

Decreased SDHB expression also could be observed in tumors associated with VHL disease (germline *VHL* mutation)^4,21^. In our study, we included two cases of hemangioblastoma associated with VHL disease and observed a weak-diffuse pattern of SDHB immunoexpression. Overall, 5~15% of tumors without *SDHx* gene mutations are interpreted as being SDHB immunonegative^21–24^. However, SDHB immunonegativity in tumors without *SDHx* gene mutations may be associated with limitations in the molecular methods or epigenetic changes^3,4,22^. Therefore, to elucidate the mechanism involved in the loss of SDHB immunoexpression in hemangioblastoma, further comprehensive molecular genetic analyses of *SDHx* mutations, including promoter methylation and/or *VHL* testing, should be performed.

## Conclusion

To the best of our knowledge, this is first study to evaluate SDHB immunohistochemistry in various types of CNS tumors. Among the CNS tumors, we found that hemangioblastoma was associated with SDHB immunonegativity, which suggests the inactivation of SDH. However, to elucidate the association between SDHB inactivation and hemangioblastoma, further comprehensive molecular analyses, including epigenetic analyses, should be conducted.

## Materials and Methods

### Patients and tumor tissues

This study was approved by the Institutional Review Board Committee of the Ajou University Medical Center (Approval No. AJIRB-BMR-OBS-16-187) and all experiments were performed in accordance with our institutional guidelines and regulations. Anonymized tissue microarray (TMA) tissue from various types of CNS tumors was used for SDHB immunohistochemistry. The surgical pathology records of all patients with hemangioblastoma between June 1994 and December 2016 were reviewed. Patients whose pathology specimens and ancillary tests were available for review were included. All slides of each case were reviewed and a representative block was selected for ancillary testing.

### Immunohistochemistry

Immunohistochemistry was performed on representative sections (4 µm thick) of formalin-fixed, paraffin-embedded (FFPE) tissues using a BenchMark XT automated immunohistochemistry stainer (Ventana Medical Systems, Tucson, AZ, USA). The Ventana staining procedure included pretreatment with a cell conditioner (pH 8) for 92 min, followed by incubation with the diluted SDHB primary rabbit polyclonal antibody (CAT# HPA002868, LOT# B105404, Sigma-Aldrich Corp; St Louis, MO, USA; 1:400) at 37 °C for 48 min. To confirm results of SDHB immunohistochemistry, immunohistochemistry using a different primary mouse monoclonal antibody against SDHB (CAT# ab14714, LOT# GR3256027-1, Abcam Inc; Cambridge, MA, USA; 1:500) was also performed. The primary antibodies were detected using an OptiView DAB IHC Detection kit (Ventana Medical Systems) following incubation with hematoxylin and a bluing reagent (4 min each). Subsequently, slides were removed from the immunostainer, washed in water containing a drop of dishwashing detergent, and mounted. The evaluation of SDHB immunoexpression was conducted by a single experienced pathologist (JH Kim) without prior knowledge of the clinicopathological data. SDHB was scored as positive if the cytoplasm showed a strong granular staining. SDHB was scored negative only if the cytoplasm was negative in parallel with positive staining for the internal control (capillary endothelial cells). Cases in which tumor cells revealed a weak cytoplasmic or nuclear blush staining without the presence of definite granular mitochondrial staining were classified as weak-diffuse pattern^22,23^.

### Mutation analyses

Genomic DNA was extracted from FFPE tumor tissues using a QIAamp DNA FFPE Tissue Kit (Qiagen, Hilden, Germany) according to the manufacturer’s instructions. DNA fragments of the SDHB gene corresponding to each exon and its flanking intron were amplified by PCR using the following gene-specific primers:

SDHB_1_F 5′-ATGCGCCGCTACTGCTACTGCGCTATT-3′

SDHB_1_R 5′-TGAGGCCTTGCCCTATGCTTCCT-3′

SDHB_2_F 5′-AATCCAGCGTTACATCTGTTGTGCCA-3′

SDHB_2_R 5′-AAGCATGTCCCTAAATCAAA-3

SDHB_3_F 5′-GAACTTTACATAAATACCACTGGA-3′

SDHB_3_R 5′-CTATCAGCTTTGGCCAGC-3′

SDHB_4_F 5′-ACCTCTGTCAGAGGAATGTTGCAT-3′

SDHB_4_R 5′-CTACTGACTAGAAGAGGAGCCTTA-3′

SDHB_5_F 5′-TGATGATGGAATCTGATCCT-3′

SDHB_5_R 5′-CAGATTGAAACAATAAATAGGGA-3′

SDHB_6_F 5′-CCTCTCTTTTCTCCCCATAC-3′

SDHB_6_R 5′-CAGCAATCTATTGTCCTCTTG-3′

SDHB_7_F 5′-AGCTAATCATCCCTGGTTTT-3′

SDHB_7_R 5′-TTGTGAGCACATGCTACTTC-3′

SDHB_8_F 5′-GTGGGTTTTCCCTTTCAGTT-3′

SDHB_8_R 5′-CGGCAAGTAAAGGAACAGGT-3′.

We also performed PCR on previously reported missense mutation sites of *SDHA* (Chr5:254599, Chr5:256509, and Chr5:223646 on Assembly GRCh37) in hemangioblastoma^30^ using the following primers:

SDHA_1F 5′-AACAGTTTGCAAGGGGAAATTACT -3′

SDHA_1R 5′-TAGATCCTTACCCCCTAAGCCA -3′

SDHA_14F 5′-GATGGTGTTTCTGGCCTCAG -3′

SDHA_14R 5′-TGTCGGAGTGCCTTTTTCAG -3′

SDHA_15F 5′-GAGAATCTTAAAGTTCACATGCC -3′

SDHA_15R 5′-GAGTGCAGAAGCGTATGAAGAC -3′

The amplified PCR products were purified and sequenced using a 3500xL Genetic Analyzer (Applied Biosystems, Foster City, CA, USA). Sequence data were compared to a reference sequence (GenBank: NG_012340.1).

### Ethical approval

This study was approved by the Institutional Review Board Committee of the Ajou University Medical Center (Approval No. AJIRB-BMR-OBS-16-187) and was performed according to our institutional guidelines and regulations (For this type of study formal consent is not required in our regulation).

## Supplementary information

Supplementary Figure

## Data Availability

All data generated or analysed during this study are included in this published article (and its Supplementary Information files).

## References

[1] Lancaster CR (2002). Succinate:quinone oxidoreductases: an overview. Biochimica et biophysica acta.

[2] Aldera AP, Govender D (2018). Gene of the month: SDH. Journal of clinical pathology.

[3] Mannelli M (2018). DIAGNOSIS of ENDOCRINE DISEASE: SDHx mutations: beyond pheochromocytomas and paragangliomas. European journal of endocrinology.

[4] Gill AJ (2018). Succinate dehydrogenase (SDH)-deficient neoplasia. Histopathology.

[5] Gottlieb E, Tomlinson IP (2005). Mitochondrial tumour suppressors: a genetic and biochemical update. Nature reviews. Cancer.

[6] Gimm O, Armanios M, Dziema H, Neumann HP, Eng C (2000). Somatic and occult germ-line mutations in SDHD, a mitochondrial complex II gene, in nonfamilial pheochromocytoma. Cancer research.

[7] Baysal BE (2000). Mutations in SDHD, a mitochondrial complex II gene, in hereditary paraganglioma. Science.

[8] Bardella C, Pollard PJ, Tomlinson I (2011). SDH mutations in cancer. Biochimica et biophysica acta.

[9] King A, Selak MA, Gottlieb E (2006). Succinate dehydrogenase and fumarate hydratase: linking mitochondrial dysfunction and cancer. Oncogene.

[10] Dahia PL (2006). & Familial Pheochromocytoma, C. Transcription association of VHL and SDH mutations link hypoxia and oxidoreductase signals in pheochromocytomas. Annals of the New York Academy of Sciences.

[11] Dahia PL (2005). A HIF1alpha regulatory loop links hypoxia and mitochondrial signals in pheochromocytomas. PLoS genetics.

[12] Letouze E (2013). SDH mutations establish a hypermethylator phenotype in paraganglioma. Cancer cell.

[13] Janeway KA (2011). Defects in succinate dehydrogenase in gastrointestinal stromal tumors lacking KIT and PDGFRA mutations. Proceedings of the National Academy of Sciences of the United States of America.

[14] McWhinney SR, Pasini B, Stratakis CA (2007). International Carney, T. & Carney-Stratakis Syndrome, C. Familial gastrointestinal stromal tumors and germ-line mutations. The New England journal of medicine.

[15] Gill AJ (2011). Renal tumors and hereditary pheochromocytoma-paraganglioma syndrome type 4. The New England journal of medicine.

[16] Ricketts CJ (2010). Tumor risks and genotype-phenotype-proteotype analysis in 358 patients with germline mutations in SDHB and SDHD. Hum Mutat.

[17] Solis DC (2009). Penetrance and clinical consequences of a gross SDHB deletion in a large family. Clinical genetics.

[18] Xekouki P (2015). Pituitary adenoma with paraganglioma/pheochromocytoma (3PAs) and succinate dehydrogenase defects in humans and mice. The Journal of clinical endocrinology and metabolism.

[19] Niemeijer ND (2015). Succinate Dehydrogenase (SDH)-Deficient Pancreatic Neuroendocrine Tumor Expands the SDH-Related Tumor Spectrum. The Journal of clinical endocrinology and metabolism.

[20] Xekouki P (2012). Succinate dehydrogenase (SDH) D subunit (SDHD) inactivation in a growth-hormone-producing pituitary tumor: a new association for SDH?. The Journal of clinical endocrinology and metabolism.

[21] Papathomas TG (2015). SDHB/SDHA immunohistochemistry in pheochromocytomas and paragangliomas: a multicenter interobserver variation analysis using virtual microscopy: a Multinational Study of the European Network for the Study of Adrenal Tumors (ENS@T). Modern pathology: an official journal of the United States and Canadian Academy of Pathology, Inc.

[22] Castelblanco E (2013). Usefulness of negative and weak-diffuse pattern of SDHB immunostaining in assessment of SDH mutations in paragangliomas and pheochromocytomas. Endocrine pathology.

[23] Gill AJ (2010). Immunohistochemistry for SDHB triages genetic testing of SDHB, SDHC, and SDHD in paraganglioma-pheochromocytoma syndromes. Human pathology.

[24] van Nederveen FH (2009). An immunohistochemical procedure to detect patients with paraganglioma and phaeochromocytoma with germline SDHB, SDHC, or SDHD gene mutations: a retrospective and prospective analysis. The Lancet. Oncology.

[25] Ni Y (2015). Germline and somatic SDHx alterations in apparently sporadic differentiated thyroid cancer. Endocrine-related cancer.

[26] Renella R (2014). Exploring the association of succinate dehydrogenase complex mutations with lymphoid malignancies. Familial cancer.

[27] Miettinen M (2014). Mapping of succinate dehydrogenase losses in 2258 epithelial neoplasms. Applied immunohistochemistry & molecular morphology: AIMM.

[28] Gladitz J, Klink B, Seifert M (2018). Network-based analysis of oligodendrogliomas predicts novel cancer gene candidates within the region of the 1p/19q co-deletion. Acta neuropathologica communications.

[29] Shiwa T (2015). A Patient with an Extra-adrenal Pheochromocytoma and Germ-line SDHB Mutation Accompanied by an Atypical Meningioma. Internal medicine.

[30] Shankar GM (2014). Sporadic hemangioblastomas are characterized by cryptic VHL inactivation. Acta neuropathologica communications.

[31] Papathomas TG (2014). Non-pheochromocytoma (PCC)/paraganglioma (PGL) tumors in patients with succinate dehydrogenase-related PCC-PGL syndromes: a clinicopathological and molecular analysis. European journal of endocrinology.

[32] Gill AJ (2014). Succinate dehydrogenase deficiency is rare in pituitary adenomas. The American journal of surgical pathology.

[33] Louis, D. N. *et al*. WHO classification of tumours of the central nervous system. Revised 4th edition. edn, (International Agency For Research On Cancer, 2016).

[34] Shin Y, Kim S, Lee HW, Bang H, Suh YL (2014). Supratentorial hemangioblastoma with unusual features. Korean J Pathol.

[35] Kaelin WG (2007). Von Hippel-Lindau disease. Annu Rev Pathol.

[36] Woodward ER, Maher ER (2006). Von Hippel-Lindau disease and endocrine tumour susceptibility. Endocrine-related cancer.

[37] Takayanagi S (2017). Differences in genetic and epigenetic alterations between von Hippel-Lindau disease-related and sporadic hemangioblastomas of the central nervous system. Neuro-oncology.

[38] Fishbein L (2017). Comprehensive Molecular Characterization of Pheochromocytoma and Paraganglioma. Cancer cell.

[39] Ma D (2017). Whole exome sequencing identified genetic variations in Chinese hemangioblastoma patients. American journal of medical genetics. Part A.

[40] Mehrian-Shai R (2016). Identification of genomic aberrations in hemangioblastoma by droplet digital PCR and SNP microarray highlights novel candidate genes and pathways for pathogenesis. BMC genomics.

[41] Bennedbaek M (2016). Identification of eight novel SDHB, SDHC, SDHD germline variants in Danish pheochromocytoma/paraganglioma patients. Hered Cancer Clin Pract.

