# Supplementary material for: The loss of succinate dehydrogenase B expression is frequently identified in hemangioblastoma of the central nervous system
Source: Sci Rep. 2019 Apr 10;9:5873. doi: 10.1038/s41598-019-42338-z (PMC6458311; doi:10.1038/s41598-019-42338-z)
Supplement: Supplementary file 1 — Supplementary Figure [file 41598_2019_42338_MOESM1_ESM.pdf]

Supplementary Information

The loss of succinate dehydrogenase B expression is frequently identified in hemangioblastoma of the central nervous system

Tae Hoon Roh[1,+], HyuneeYim[2,+], Jin Roh[2], Kyi Beom Lee[2], So Hyun Park[2], Seon-Yong Jeong[3], Se-Hyuk Kim[1,*], Jang-Hee Kim[2,*].

[1] Department of Neurosurgery, Ajou University School of Medicine, Suwon, 16499, Korea

[2] Department of Pathology, Ajou University School of Medicine, Suwon, 16499, Korea

[3] Department of Medical Genetics, Ajou University School of Medicine, Suwon, 16499, Korea

Contents

Supplemental Figure S1. SDHB and SDHA immunohistochemistry in hemangioblastoma.

Supplemental Figure S2. HIF-1α expression in hemangioblastoma with a weak-diffuse pattern of SDHB immunostaining.

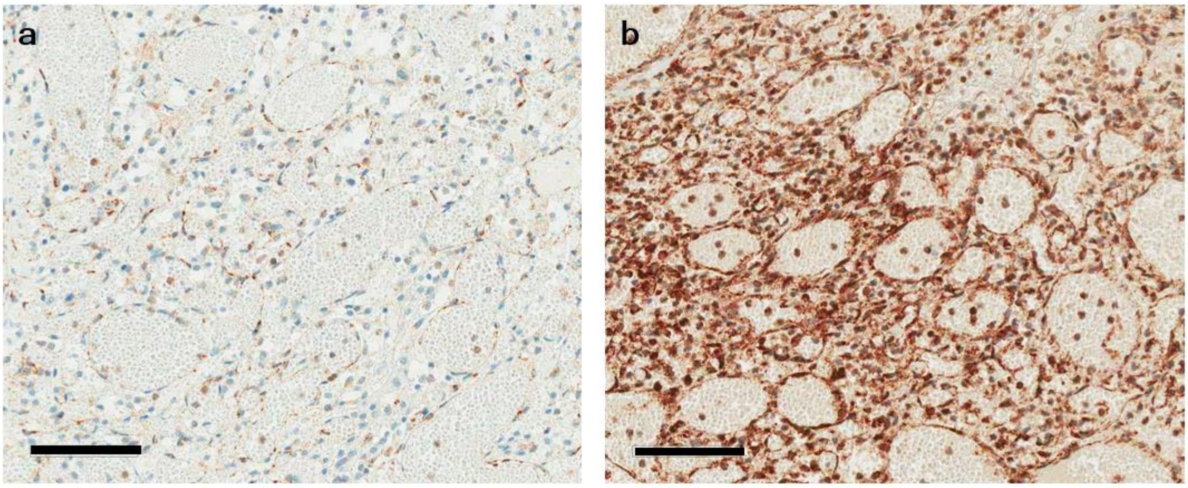

Supplementary Figure S1. SDHB and SDHA immunohistochemistry in hemangioblastoma. (a) Negative SDHB immunostaining in the stromal tumor cells and (b) Positive SDHA immunoexpression at the corresponding area of hemangioblastoma. Bar indicates 100 μm.

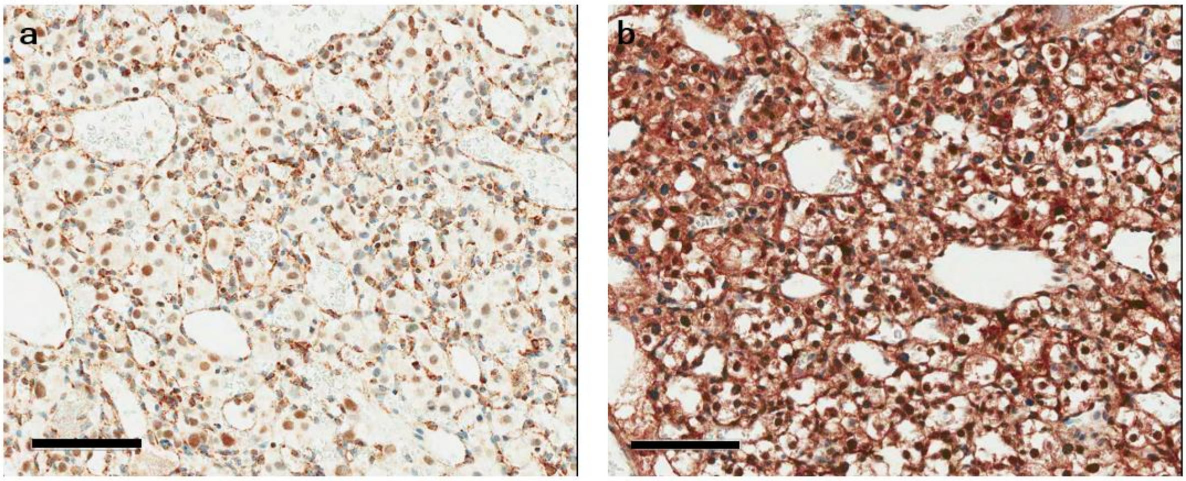

Supplementary Figure S2. HIF-1α expression in hemangioblastoma with a weak-diffuse pattern of SDHB immunostaining. (a) a weak-diffuse pattern of SDHB immunostaining (b) HIF-1α immunostaining in the corresponding areas. Increased expressions of HIF-1α are noted at the nuclei and cytoplasms of the stromal tumor cells in hemangioblastoma. Bar indicates 100 μm.
